# Retention rate in methadone maintenance treatment and factors associated among referred patients from the compulsory residential centers compared to voluntary patients

**DOI:** 10.3389/fpsyt.2023.1139307

**Published:** 2023-05-25

**Authors:** Niayesh Radfar, Seyed Ramin Radfar, Faezeh Mohammadi, Amir Azimi, Ali Amirkafi, Arash Tehrani-Banihashemi

**Affiliations:** ^1^Department of Medicine, Isfahan University of Medical Sciences, Isfahan, Iran; ^2^Department of Neuroscience and Addiction, School of Advanced Technologies in Medicine (SATiM), Tehran University of Medical Sciences, Tehran, Iran; ^3^Integrated Substance Abuse Programs Department, University of California, Los Angeles, CA, United States; ^4^Preventive Medicine and Public Health Research Center, Psychosocial Health Research Institute, Iran University of Medical Sciences, Tehran, Iran; ^5^Department of Medicine, Iran University of Medical Sciences, Tehran, Iran

**Keywords:** compulsory treatment, volunteer treatment, methadone maintenance treatment, retention rate, Iran

## Abstract

**Introduction:**

Compulsory treatment has decades of history in Iran; both before and after the Islamic Revolution, but there are many debates regarding its efficacy and effectiveness. Retention Rate is one of the best indices to estimate the efficacy of treatment. This study will compare Retention Rate among people referred from compulsory treatment centers and volunteer participants.

**Methods:**

This was a retrospective (historical) cohort study that has been conducted among people who were taking methadone maintenance treatment (MMT). The study sample was selected from the MMT centers that admit both referral patients from compulsory centers and voluntary patients. All newly admitted patients from March 2017 to March 2018 were enrolled and followed up until March 2019.

**Results:**

A total of 105 participants were recruited for the study. All were males with a mean age of 36.6 ± 7.9 years. Fifty-six percent of individuals were referred from compulsory residential centers. The total one-year retention rate of participants in this study was 15.84%. The one-year retention rate for the patients referred from compulsory residential centers and the non-referred patients was 12.28 and 20.45%, respectively (value of *p* = 0.128). Among the other studied factors, only marital status was significantly associated with MMT retention (*p* = 0.023).

**Conclusion:**

Although the average treatment adherence time for non-referred patients was about 60 days higher than those referred from compulsory residential centers, this study found no significant differences in retention days and a one-year retention rate. Further studies with larger sample sizes and longer follow-ups are needed to explore the efficacy of compulsory treatment methods in Iran.

## 1. Introduction

One of the greatest challenges for various parts of the community, including the judicial system, law enforcement, and undoubtedly the health system, is substance use around the world. In Iran, substance use accounts for a significant portion of the Disability Adjusted Life Years (DALY) and the Years Lived with Disability (YLD) index (1). According to estimations, Iran’s DALY index and YLD index are 698 per 100,000 people and 11.1 (2), respectively. The severity of the issue in Iran is evidenced by comparing with the average number of these indices in the world, which is 200 per 100,000 people and 4 per 100,000 (3).

Iran moved from conservative and criminalization programs towards a more liberal approach for medicalized drug addiction treatment and harm reduction with consequential drawbacks during the last 40 years after the Islamic Revolution. Contradictory liberal and conservative addiction treatment programs exist simultaneously in Iran’s current drug addiction treatment scene (4).

Since roughly 17 years ago (2003–2004), a significant network of outpatient substance use treatment clinics has been established in Iran. Currently, there are about 7,000 clinics with about 700,000 patients undergoing opioid substitution therapy (mostly methadone maintenance therapy) in the country (5).

Other than traditional opioid use among Iranian substance users, another substance use disorder that increased in the first decade of the new century was methamphetamine use. Methamphetamine seizures were reported in Iran for the first time in 2005 and scaled up as fast as that, putting Iran fifth in the world in 2010–2011 based on the number of methamphetamine seizures (6). It is essential to mention that methamphetamine use among the patients undergoing MMT is also common and many patients started using methamphetamine to cope with methadone side effects such as sexual dysfunction as well as lethargy and drowsiness (7, 8). One study reported that nearly 90% of female patients taking methadone from the MMT clinics were using methamphetamine to some degree simultaneously (9). In response to this situation, a guideline for the integration of methamphetamine harm reduction interventions into the ordinary harm reduction services for opioid users was developed by the ministry of health with support of the UNODC, Tehran (10).

Unfortunately, the issue still exists despite this extensive network of treatment centers and an almost adequate network of services and facilities for harm reduction, including Drop-in Centers (DIC), outreach teams, and hundreds of midterm residential campuses. Following an increased number of people with problematic substance use disorders, and public and political demand for more strict policies towards drug use, compulsory treatment programs which were on the path to decline, were scaled up again; especially after the emergence of the methamphetamine crisis.

People who use drugs (PWUD) were given the option of being sentenced to compulsory treatment if they refused to participate in a treatment program or harm reduction service or caused a public nuisance under a new amendment to the drug control law that took effect in 2010 (Iran Council of Expediency 2010, article 16). These compulsory rehabilitation centers offer OST or abstinence-based programs in a residential setting for a few months and are designed to be formally governed in a court-based setting. People who receive OST services later contact DICs to continue receiving maintenance treatments at a partial or full subsidized cost. Compulsory treatment has more than 50 years of history, as we can trace the first experiences back to 1961 in the United States (11). Iran also has a history of compulsory medical intervention both before and after the Islamic Revolution. There are still numerous arguments over its efficacy. Retention rate, one of the indicators that can be used to gage the effectiveness of the treatment, is impacted by a variety of circumstances (12–15).

Although some scientists made efforts to justify compulsory treatment with the rationale that long-lasting changes in the brain, hijack a person’s ability to refrain from drug use, and in this condition, others are allowed to put patients in a compulsory rehabilitation centers, some studies showed these models might have negative consequences for the patient rather than being effective (16). It is necessary to consider that there are many debates against compulsory treatment. i.e., a review evaluated the clinical effectiveness of compulsory treatment, and concluded that current evidence does not support the idea that compulsory treatment modalities are effective for drug dependence treatment. Some studies even suggest it to be harmful (17, 18).

In the Lancet Global Health, Martin Wegman and colleagues present their study of opioid use in opioid-dependent individuals released from compulsory drug detention centers (CDDCs) compared with those from voluntary methadone treatment centers (VTCs) in Malaysia. This study was the first prospective observational study to compare drug-use outcomes between the two facility types. The investigators showed that opioid-dependent individuals in CDDCs were significantly more likely to relapse to opioid use after release than opioid-dependent individuals receiving methadone in VTCs (in unadjusted analyses, CDDC participants had a significantly more rapid relapse to opioid use post-release compared with VTC participants [median time to relapse 31 days (IQR 26–32) vs. 352 days (256–inestimable), log-rank test *p* < 0·0001) (19)].

Studies in Iran found some factors influencing retention rates in treatment, such as methadone dose, polysubstance use, being treated in private or governmental clinics, (20), stimulant drug use, comorbidity disorders (21), distance to a clinic, perceived social support, and perceived pleasure with drug use (22). A qualitative study also revealed that treatment cost and family support are among the factors that can influence the retention rate in treatment (23).

Although the recent wave of compulsory treatment in Iran has lasted for nearly a decade, only a few independent studies have evaluated the effectiveness of these models compared to the voluntary-based modalities. To our knowledge this is one of the first studies in this field in Iran that has tried to find the retention rate of patients referred from compulsory treatment centers compared to those who are coming by their own will.

## 2. Materials and methods

### 2.1. Study design

This study was a retrospective cohort study. The study population was made up of patients who were taking methadone maintenance treatment (MMT) for the treatment of drug use. The study sample was selected from the ‘Haftoon’ MMT center. This center receives both volunteer individuals in addition to referred patients from compulsory rehabilitation centers in the city of Isfahan, Iran. Eligibility criteria include (1) age more than 18 years, and (2) being qualified for MMT based on the ministry of health protocol for MMT which recommend MMT for patients with one of these criteria:

- Current heroin user
- Opioid users with a history of injection of any drug
- History of two-time failed abstinence treatment for opioid users
- People living with HIV

and (3) referred from the judicial system and/or voluntarily admitted patients for MMT.

All newly admitted patients during the years 1,396 and 1,397 (March 21st, 2017 to March 20th, 2018) in the mentioned center were enrolled in the study and were followed up until March 20th, 2019. Patients whose follow-up data were missing and their treatment status was unclear were excluded from the study. The National Institute approved this study for the Medical Research Development (NIMAD) review board, and the following ethical approval code was granted: IR.NIMAD.REC.1398.123.

### 2.2. Data sources and measures

The study utilized the physical files, which are typically used for routine clinical and program monitoring. A researcher made questionnaire was used to collect demographic, drug history, mental health, and HIV risk behavior data, for each MMT client. Demographic data included age (in years), education level (primary-level schooling or less/more than primary-level schooling) and marital status (currently married/not married); Injection-related risk factors included shared needle use at last injection, shared other equipment at last injection, cleaned needles with bleach if sharing at the last injection, and poly-substance use (heroin and alcohol, cocaine, benzodiazepines, or amphetamine). Date of entry, possible date of drop-out, methadone daily dose, and days spent in compulsory rehabilitation centers were acquired from patient files.

Retention was defined as the number of days from entering the study to the end of the study or leaving the treatment. Two weeks of missed appointments were considered a drop-out for patients.

### 2.3. Statistical methods

Descriptive measures were used to describe the patients’ characteristics, including frequencies, mean, and standard deviation. The life tables and the Kaplan–Meyer method were used to calculate the retention rate for each group and compare between groups; the Cox proportional hazard model was used to find the factors associated with retention rate. The Hazard Ratio and 95% Interval for each variable are presented. Also, the level of significance was set at 0.05. All the analysis was carried out by SPSS software version 26.

## 3. Results

### 3.1. Study characteristics

A total of 105 patients were recruited; due to incomplete follow-up data, four were excluded from the study. All 101 participants were male, and 56.4% were referred from compulsory residential centers. Upon entry, they had a mean age of 36.5 years (SD = 7.9 years), and the age range was from 20 to 65 years. Nearly half were married (50.5%), and only 5% were HIV positive. Most of them used heroin (10.9%), and smoking was the most common route for substance use among them (11.9%). At the time of admission, the mean dose was 50.1 ± 18 mg, and the mean of the final dose that participants received was 61.5 ± 29. Analysis showed no difference in demographics except for the admission dose of methadone, which was higher in the referred group. Demographic characteristics are available in Table 1.

**Table 1:** Demographic characteristics of participants.

		General		Referred from compulsory centers		Volunteer group		Value of *p*
Variable	Groups	Number	Percentage	Number	Percentage	Number	Percentage	
Referral	Yes	57	56.4					
	No	44	43.6					
Age	<30	21	20.8	15	26.3	6	13.6	0.053
	31–40	53	52.5	32	56.1	21	47.7	
	41–50	21	20.8	9	15.8	12	27.3	
	>50	6	5.9	1	1.8	5	11.4	
Marital status	Single	50	49.5	29	50.9	21	47.7	0.754
	Married	51	50.5	28	49.1	23	52.3	
HIV status	Negative	96	95	56	98.2	40	90.9	0.092
	Positive	5	5	1	1.8	4	9.1	
Main drug used	Heroin	11	10.9	11	19.3	-	-	-
	Opium Juice	1	1.0	1	1.8	-	-	
	Opium	1	1.0	1	1.8	-	-	
	Crack	1	1.0	1	1.8	-	-	
	Missing	87	86.1	43	75.4	44	100	
Main route of administration	Smoking	12	11.9	11	19.3	1	2.3	0.875
	Injection	2	2.0	2	3.5	-	-	
	Oral	1	1.0	1	1.8	-	-	
	Missing	86	85.1	43	75.4	43	97.7	
Admission dose	<50	41	40.6	15	26.3	26	59.1	0.002
	> = 50	60	59.4	42	73.7	18	40.9	
Final dose	<50	27	26.7	12	21.1	15	34.1	0.814
	> = 50	74	73.3	45	78.9	29	65.9	

### 3.2. Retention rate

The total median of retention days was 92 days; 80 (79.2%) of participants did not adhere to treatment; the median of retention in non-adherent patients was 61.5 days, and the mean was 105.6 ± 108.9 days.

**Figure 1:**
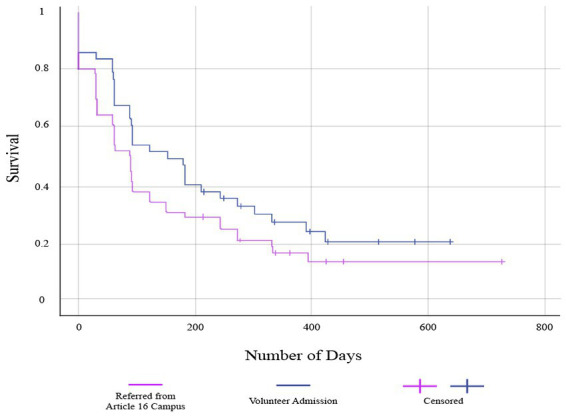
Retention rate among volunteers and patients referred from compulsory treatment centers.

The median retention days for referred patients were 90 days, and for non-referred patients, it was 153 days. The life tables and the Kaplan–Meier method were used to calculate and compare retention durations totally and, in each group, separately. The retention rate between the two groups was not significantly different (*p*-value = 0.125; Figure 1). Six-month and one-year retention rates among participants receiving MMT voluntarily were higher compared to the referred patients (value of *p* = 0.093 & 0.128, respectively; Table 2).

**Table 2:** 6 Months and 1 year retention rate.

	Retention rate %			*p*-value
	Total	Referred from compulsory centers	Volunteer group	
6 months	39.60	31.57	50.0	0.093
1 year	15.84	12.28	20.45	0.128

### 3.3. COX regression

Being referred from compulsory rehabilitation centers, age, marital status, admission dose, final dose, and HIV status were entered into the model; among them, only marital status was significantly associated with MMT retention, as married participants were more likely to adhere to treatment (*p* = 0.023); detailed associations with all variables for both referral groups are mentioned in Table 3.

**Table 3:** Cox’s proportional hazards model analyses on factors associated with retention duration.

Variable	Groups	Article 16 referred group		Volunteer group		total	
		HR	95% CI	HR	95% CI	HR	95% CI
Referred from compulsory centers	Yes	Ref					
	no	-	-	-	-	1.39	0.89–2.18
Age group	20–29	Ref					
	30–39	0.76	0.36–1.60	0.79	0.24–2.96	0.74	0.39–1.38
	>40	0.73	0.31–1.73	0.73	0.22–2.82	0.66	0.34–1.32
Marital status	Single	Ref					
	Married	1.90*	1.05 – 3.45	1.33	0.67–2.65	1.67*	1.07 – 2.61
Admission dose	<50	Ref					
	> = 50	1.61	0.98–2.92	0.73	0.31–1.69	1.32	0.84–2.05
Final dose	<50	Ref					
	> = 50	0.79	0.44–1.44	2.0	0.99–4.03	1.17	0.75–1.83
HIV status	positive	Ref					
	negative	0.04	0.0–189	0.40	0.09–1.7	0.30	0.07–1.23

* *p*-Value < 0.05.

## 4. Discussion

In this study, we aimed to compare retention rates in voluntary and compulsory MMT treatment, which is an essential step in determining the effectiveness of drug detention (24). The results showed that the median retention days for the referred patients were lower than for voluntary patients (90 days vs. 153 days) but this was not statistically significant, which could be due to small sample size. Also marital status was the only factor affecting the retention, and married participants had higher retention duration.

The total one-year retention rate of participants in this study was 15.84%. A systematic review of 63 observational studies of opioid substitution treatment reported a 57% retention rate in 1 year (25). One of the reasons for this difference could be that the study mentioned above included MMT, buprenorphine, and mixed substantive opioid treatments, whereas our study only included MMT. In our study, the mean final dose of methadone was 61.5 mg/day, which was lower than in similar studies. Methadone dose has been shown to be an important factor in retaining patients on treatment. Likewise, in heroin users, doses higher than 80 mg/day predicted higher retention (26–28). The low-dose methadone also could be one of the other reasons for the lower retention rate in our study, which must be addressed in an appropriate manner by the responsible authorities considering the risk of diversion and overdose. It is good to mention that in another study that had been conducted in Iran, the average daily dose was 57.06 ± 18.04 mg/day, and the higher dose was known to have higher chance of one-year retention in the treatment (20).

The ethical dilemma of forced addiction treatment is complex from a public health perspective. Substance use disorders create a real threat to public health, and give governments and other formal institutions a justification to intervene in the lives of people who use drugs (PWUD). It must also be considered whether the benefits outweigh any negative consequences resulting from violating the individual’s right to make their own decisions about treatment. it is not clear whether the exposure to compulsory treatment is ultimately beneficial or harmful in the long term for the individual and for the public (29).

People in voluntary MMT have a stronger sense of ownership over their treatment and, consequently, are more likely to stay longer on treatment (30). In contrast, it is believed that those referred from compulsory residential centers may be less motivated to continue treatment after being released from the detention center. A study comparing the readiness to change and treatment in voluntary and compulsory treatment showed that although the compulsory patients scored significantly lower levels of motivation than the voluntary patients, both the compulsory and the voluntarily admitted patients were mostly at the highest level of readiness to seek help on admission and discharge, and that the change readiness stage at admission did not predict retention in treatment (31). In our study, the level of motivation was not assessed, but the results showed that the treatment of voluntary participants and those referred patients was equally effective in retaining patients. A cross-sectional study in China showed that occupation, family support, and social function equally increased in both voluntary and compulsory drug use rehabilitation centers after treatment (32).

In this study patients referred from compulsory residential centers had a higher admission dose but the final dose in the two groups was not significantly different. The decision for the initial dose is based on the clinical evaluation of the patient’s condition, the type and amounts of illicit drug use, and opioid tolerance (33); so this difference could be due to different types and amounts of previous drug use. The data about the type of drug used is missing in the voluntary group and we are not able to compare patients referred from compulsory treatment and voluntary patients in this category. The analysis of other demographic characteristics showed no other significant difference between these two groups. Among referred patients, heroin and other opioids were the most common type of drug used. A study in residential drug treatment centers in Iran showed that 72.5% of the patients were opioid users (34), also the rapid situation assessments in Iran almost always found that opioid substances are in the top of list among Iranian PWUD; which shows the importance of MMT treatment in Iran (35–37).

G.M. Heyman evaluated age of onset for substance use and explored that the most of PWUD in the US stop using illegal drugs around the age 30, due to legal concern, economic pressure, and desire for respect from their family members (38). Another qualitative study in Iran found that stigma from the family plays an important role for treatment adherence among the People living with HIV (39). In parallel with these findings, among the factors evaluated in this study only marital status significantly correlated with the duration of retention in MMT and married participants were more likely to stay on treatment. A systematic review found age, marital status, employment status, and gender to be positively associated with adherence to treatment with methadone (25). In our study, all of the included patients were male and employment status was not evaluated. The effect of age was not statistically significant in our study.

## 5. Conclusion

This study showed no significant difference in the retention rate between patients referred from the compulsory residential centers and those who voluntarily joined MMT treatment. Among the factors assessed in this study, only marital status significantly correlated with the retention rate —married patients stayed longer in treatment.

## 6. Limitations

This study was conducted at a single center over a period of 1 year. Designing a multi-center study with a longer data collection time will help to get a better understanding of the effectiveness of compulsory treatment in Iran. Data about the type of illicit drug used was missing in the voluntary treatment group, making it impossible for us to assess its effect on treatment retention and to compare the two groups. Illicit drug use during MMT treatment could be one of the confounding factors. Future studies can use urine toxicology results to compare and control the effects of illicit drug use in compulsory and voluntary participants.

## Data availability statement

The raw data supporting the conclusions of this article will be made available by the authors, without undue reservation.

## Ethics statement

This study was approved by the National Institute for Medical Research Development (NIMAD) review board and the following ethical approval code was granted: IR.NIMAD.REC.1398.123. The patients/participants provided their written informed consent to participate in this study.

## Author contributions

For this study, AT-B and RR worked on the idea and design of the study. RR and NR gathered and organized the data. AAm performed the statistical analysis. AAz and FM wrote the first draft of the manuscript. All authors contributed to the article and approved the submitted version.

## Funding

This work was supported by the “National Institute for Medical Research Development” under Grant number 977086.

## Conflict of interest

The authors declare that the research was conducted in the absence of any commercial or financial relationships that could be construed as a potential conflict of interest.

## Publisher’s note

All claims expressed in this article are solely those of the authors and do not necessarily represent those of their affiliated organizations, or those of the publisher, the editors and the reviewers. Any product that may be evaluated in this article, or claim that may be made by its manufacturer, is not guaranteed or endorsed by the publisher.
